# Checkpoint Inhibitor-Induced Colitis—A Clinical Overview of Incidence, Prognostic Implications and Extension of Current Treatment Options

**DOI:** 10.3390/ph14040367

**Published:** 2021-04-16

**Authors:** Carmen Portenkirchner, Peter Kienle, Karoline Horisberger

**Affiliations:** 1 Department of Surgery, Cantonal Hospital of Baden, 5404 Baden, Switzerland; Carmen.Portenkirchner@ksb.ch; 2 Department of Abdominal and Transplantation Surgery, University Hospital of Zurich, Rämistrasse 100, 8091 Zürich, Switzerland; 3 Department of General and Visceral Surgery, Theresienkrankenhaus and St. Hedwig-Klinik GmbH, 68165 Mannheim, Germany; p.kienle@theresienkrankenhaus.de

**Keywords:** immune checkpoint inhibitor, immune checkpoint inhibitor-induced colitis, colitis, irAE, IBD, resumption, fecal transplantation, ileostomy

## Abstract

In recent years, anti-tumor immunotherapies have witnessed a major breakthrough with the emergence of immune checkpoint inhibitors (ICIs). However, the use of ICIs has also brought an era of a certain class of adverse events that differ from those of classical chemotherapies and are more reminiscent of autoimmune diseases. This article focuses exclusively on colitis as an irAE with emphasis on vulnerable patient groups, the prognostic significance of colitis, treatment, and new therapeutic approaches that may be applicable. Colitis itself is associated with a favorable oncological outcome of the underlying disease but is as well the most common irAE leading to discontinuation of therapy. Especially in vulnerable patient groups such as IBD patients and elderly patients, colitis occurs more frequently as a side effect. It is precisely in these two patient groups that side effects more often lead to discontinuation of therapy. Therefore, in addition to the current therapy of colitis through immunosuppression, the focus should also be on new forms of therapy of severe colitis, such as fecal transplantation or ileostomy creation.

## 1. Introduction

In recent years, anti-tumor immunotherapy has witnessed a major breakthrough with the emergence of immune checkpoint inhibitors (ICIs). Monoclonal antibodies targeting programmed cell death protein 1 (PD-1), programmed death ligand 1 (PD-L1), and cytotoxic T-lymphocyte-associated antigen 4 (CTLA-4), alone or in combination, show anti-tumor activity by enhancing the immune system via T-cell activity and inhibiting the inhibition of the immune system [1,2]. The anti-tumor action of ICIs is therefore mediated by enhancing the anti-tumor capabilities of T-cells, rather than direct cytotoxicity against the tumor [3,4,5]. This broad principle of action makes use of the drugs possible in a vast number of tumors. Indeed, ICIs have quickly become standard therapies for many diseases, as they have the capability to ameliorate even highly advanced disease conditions, leading to a significant improvement in overall survival and/or progression-free survival in a variety of malignancies [6,7,8,9,10].

However, the use of ICIs is also associated with the development of a particular class of adverse events that differ from those of classical chemotherapies and that are more reminiscent of autoimmune diseases. These side effects caused by an unregulated immune reaction are collectively named “immune-related adverse events” (irAE). These irAEs can affect a wide range of organs, have varying degrees of severity, and range widely in the timing of onset. They usually occur after a relatively short period of therapy but can also occur very late or even after treatment has been discontinued. The lower gastrointestinal (GI) tract is often affected, which may become clinically apparent through the development of diarrhea and even colitis (Table 1) [11,12]. It is now evident that the risk of an adverse event differs markedly between drug classes, with colitis occurring less frequently after PD-L1 inhibitors than after CTLA-4 drugs and most often when the two agents are used together [13].

Furthermore, it is likely that the incidence of colitis, like all irAEs, depends on both the particular ICI drug used and also on the patient. How elderly patients and those with inflammatory bowel disease (IBD) respond to ICI therapy needs to be specifically considered.

The more the immune processes are understood and reliably trackable by biomarkers, the more that toxicity will be preventable. Biomarkers, yet to be used clinically, are likely to become an additional factor to be considered in daily clinical decision-making [15].

The therapeutic approach to irAEs involves increasing levels of immunosuppression to counteract the increased immune activity caused by the active substance of the ICI. However, colitis in particular, especially if severe, may not be manageable by medication alone, and may require surgical therapy, similar to colitis due to IBD.

This article focuses exclusively on colitis as an irAE with emphasis on vulnerable patient groups, the prognosis and whether new therapeutic approaches may be beneficial.

## 2. Physiology of CTLA-4 and PD-1/PD-L1Activity

The mobilization of quiescent T-cells is a sophisticated and compound process that involves intracellular signaling. It is initiated by the T-cell antigen receptor and then further modulated and counterbalanced by costimulatory and inhibitory receptor signaling [16].

CTLA-4 is known to reduce the extent of the immune response [17]. However, the specific pathways by which CTLA-4 restricts T-cell activation have not yet been completely clarified [18,19]. Naïve and resting memory T-cells appear to express stimulatory receptors on the surface, consistent with the initial response to antigen recognition. Studies show that CTLA-4 is mobilized from intracellular protein stores to the cell surface so rapidly that feedback inhibition can occur within an hour of antigen contact [20]. The importance of this is demonstrated by the significant mortality of CTLA-4 knockout mice, with an autoimmune mechanism appearing to contribute [18,19,20,21,22]. PD-1 is not expressed on resting naïve and memory T-cells; it is only expressed after T-cell receptor activation [19]. This counts the same for CTLA-4. However, PD-1 expression on the surface of activated T-cells implies transcriptional activation and is therefore delayed [16,19]. Moreover, the effects of knockouts of PD-1 and PD-L1 in the murine models are mild and demonstrate late-onset organ-specific inflammation [19,23]. This is in accordance with the clinical picture, as autoimmune side effects of anti-PD-1 drugs are milder and occur less frequently than with anti-CTLA-4 drugs [19].

### Anti-Tumor Immunity

CTLA-4-mediated suppression of anti-tumor immunity is thought to occur primarily in secondary lymphoid organs, where T-cell activation also occurs [18]. In contrast, PD-1 blockade is thought to act predominantly in the tumor microenvironment, where its ligands are frequently overexpressed by both tumor cells and infiltrating leukocytes [19,24].

While PD-1 signaling reduces the function of activated T-cells in the periphery, CTLA-4 signaling already inhibits T-cell activation and thereby maintains immune tolerance. In fact, although CTLA-4 antibodies appear to act more causative, PD-1-blocking antibodies have shown better tolerability and potentially better efficacy.

## 3. Pathophysiology of ICI-Induced Colitis

In addition to down-modulating the activity of helper T-cells, CTLA-4 also enhances the immunosuppressive activity of regulatory T-cells (Treg). Blocking CTLA-4 therefore leads to broad activation of immune responses that are dependent on helper T-cells and inhibits Treg cell-dependent immunosuppression [25].

Since it is likely that also cross-reactive T-cells (i.e., T-cells that bind to both tumor and irAE target tissues) and tumor-specific T-cells are affected and activated by these processes, it is easy to appreciate that immune-related side effects may also occur [26]. Accordingly, many irAEs are characterized by cellular autoimmunity with T-cells and histiocytes in the target tissues [26]. Case studies indicate that specific antigenic targets can drive anti-tumor responses and organ-specific autoimmunity [26,27,28]. The clinical correlation between tumor response and the occurrence of irAE will be focused on later in this review.

The exact role of these cross-reactive T-cells in the pathophysiology of irAE is still unclear [26].

Some studies describe the risk of ICI-induced colitis as at least partly dependent on the gut microbiome [26,29,30]. In fact, the diverse microbiome appears to influence not only the development of irAE but also the tumor response to ICI therapy [31].

### 3.1. Histopathological Features of Colitis Induced by Immunotherapy

The histological features are largely similar to those of acute colitis [32,33]. Cryptitis, intra-epithelial neutrophilic lymphocytes, mucosal ulcerations, crypt abscesses and apoptosis are common features in ICI-induced colitis [34,35,36,37,38]. A study of patients with ICI-induced colitis of grade 2 or higher showed that inflammatory changes, such as foci of neutrophilic cryptitis, crypt abscesses, glandular destruction, and erosions of the mucosal surface were diffusely displayed in three quarters of the cases [32]. However, there seems to be a lack of correlation between these characteristics and the degree of diarrhea [36,37]. Data on the incidence of chronic colitis are contradictory [32,34,36,38].

Microscopic colitis (lymphocytic colitis and collagenous colitis) has been described in just over 10% of ICI-induced colitis [33].

### 3.2. Comparison to Inflammatory Bowel Disease

There are very few studies comparing the histopathological characteristics of ICI-induced colitis to IBD colitis [35,39]. However, the two entities definitely appear to be histopathologically distinct from each other [35,39]. While apoptosis is described inconsistently in IBD colitis, it is a common histopathological feature in ICI-induced colitis [35,39]. Cryptitis and mucosal ulceration both seem to occur with approximately equal frequency in both syndromes [35,39]. A study that compared anti-CTLA-4-induced colitis and ulcerative colitis (UC) showed a higher incidence and greater severity of crypt distortion in the former than in UC [35]. Interestingly, this study also showed a higher number of B-lineage cells in UC compared to the anti-CTLA-4-induced colitis group. This observation supports the notion that pathogenesis in ICI-induced colitis is predominantly driven by T-cells, whereas in this and previous studies humoral immunity was shown to be more important in IBD [35,40].

Another finding suggesting a fundamental difference in IBD and ICI-induced colitis is that serum from patients with anti-CTLA-4-induced colitis has fluctuating levels of antibody titers against microbial flora while these titers are characteristically altered in classic IBD [32,35,41].

### 3.3. Biomarkers

Biomarkers may be of value in preventing possible overtreatment of ICIs or in improving early detection of an irAE. This could contribute to a more effective treatment. However, the term biomarker is quite broad and can encompass anything from gender, age or tumor type to molecular markers. Even though individual studies show promising correlations between IL-17 or IL-6 and development of an irAE, for example, these analyses have not yet been implemented in clinical practice [42,43,44].

It would be advantageous if the individual risk of developing an irAE could reliably be identified by serum tests. To date, however, there are no good predictive biomarkers available [44]. As the immune response is such a complex process, the identification of specific biomarkers remains difficult. The identification of reliable biomarkers would be a further step towards better understanding of the immune processes as a response to ICI therapy and therefore needs be pursued further [44]. Most importantly, with the aid of reliable markers, better adaptation and guidance of treatment would be possible.

## 4. Incidence

Development of colitis in patients treated with anti-PD-1 or PD-L1 agents occurs with a rate of 0.0–1.4%, and colitis of grade 3 or higher occurs in 0.0–0.9%, according to two systematic reviews [45,46]. With CTLA-4 therapy, the rate of colitis is reported to be 5.7–9.1%, with an incidence of grade 3 colitis of 4.1–6.8% [45,46]. With ipilimumab, a dose-dependent incidence of colitis can be observed [47]; no dose-dependent incidence of colitis has been documented with the other ICIs.

In most studies, a combination of different ICIs results in the highest rates of colitis; this holds true for the combination of durvalumab and tremelimumab [48], and the combination of nivolumab and ipilimumab (Supplementary Tables) [49,50,51]. The overall rate of colitis and especially severe colitis (grade 3–4) is well above 10%. The combination of ipilimumab and nivolumab before or after chemotherapy has a higher complication rate than monotherapy, with the rate almost twice as high when the ICI combination is given after chemotherapy [49]. However, the highest rates are seen with sequential administration of nivolumab and ipilimumab, and in particular with the administration of nivolumab after ipilimumab [50]. In a recent retrospective study using a combination of nivolumab and ipilimumab, 32% of the patients developed colitis, with 11.2% of patients developing grade 3 colitis [52].

A meta-analysis of clinical trial data consisting of 3721 patients from 30 study arms showed a 27% rate of colitis (95% CI 19–34%) when anti-CTLA-4 ICIs were used (20 CTLA-4 therapy study arms, 10 combined PD-1/PD-L1 and CTLA-4 therapy study arms), and 58% (95% CI 41–76%) of these had colitis of grade 3–5 in severity. In contrast, only 11% (95% CI 7–15%) had colitis when receiving combination therapy with PD-1/PD-L1 and CTLA-4, of which only 15% (95% CI 8–22%) were of a more severe grade (grade 3–5) [53].

The risk of colitis appears to depend on the underlying malignancy. The rates of colitis in patients treated with anti-PD-1/PD-L1 ICIs were higher if the patients had advanced melanoma than in patients with advanced lung cancer or renal cell cancer. The cause of this difference is not clear [46].

The influence of the patient’s characteristics, independent from the underlying malignancy, on the risk of developing, and the magnitude of, ICI-induced colitis cannot currently be adequately assessed, due to the paucity of available data. For example, nothing is yet known regarding a gender-specific risk of developing colitis.

In a multivariate logistic regression model, the relationship between drug class, dose, and underlying disease on the development of colitis was investigated in a review [42]. Only the drug class showed a significant correlation [45]. The risk of developing colitis was more than three times higher with anti-CTLA-4 therapy than with PD-1/PD-L1 inhibitors (OR 3.12; 95% CI 1.06–9.24) [45].

### 4.1. Correlation to Response

It is becoming more apparent that a favorable response to ICI therapy is associated with an increased risk of an adverse event. However, patients with rapidly progressive disease may be less likely to experience an irAE because they may die before experiencing one, which may make the strength of the association less robust. For this reason, some studies only included patients with a minimum follow-up in their analyses and could indeed show a correlation, with no specific reference to a particular irAE [54,55,56,57,58]. A systematic review was also able to demonstrate the same finding [59]. Based on individual irAEs, a correlation has been shown for colitis [60,61,62] as well as for cutaneous adverse events and pneumonitis [63,64,65,66,67,68,69].

### 4.2. Discontinuation of Therapy

Discontinuation of treatment with an ICI due to development of an irAE is often not reported in trials; when reported, discontinuation rates were in the range of 3–12% in anti-PD-1 trials and 3–25% in anti-CTLA-4 trials. The most common irAEs leading to discontinuation were diarrhea and colitis [45].

Deaths under ICI therapy are rare, but mostly occur due to irAE. A systematic review examined the causes of death [45]. In anti-CTLA-4 trials, the mortality rates were approximately 1%; one third of these were deaths following GI events, especially colitis, and colonic perforation. With PD-1/PD-L1 inhibitors, deaths were exceptionally rare [45].

### 4.3. ICI-Induced Colitis in Pre-Existing Inflammatory Bowel Disease

One major problem regarding the assessment of the risk of a flare-up of colitis in IBD patients receiving ICI therapy is that such patients were excluded from the prospective studies on ICI. However, several cohort analyses exist of patients with different autoimmune diseases who were treated with ICIs (Table 2). The largest study to date, with 112 patients, included only 14 IBD patients [70]. Of these, 100% experienced a flare-up of IBD when receiving ICI therapy [70]. The largest study containing only IBD patients included a retrospective multicenter analysis of 102 patients; 22% were receiving immunosuppressive therapy [71]. The patients had Crohn’s disease (CD) or ulcerative colitis (UC) in equal proportion. Forty-one percent of the patients had a GI event after 62 days (IQR 33–123 days) [71]. Four patients suffered from colonic perforation (two CD, two UC), but no GI toxicity-related deaths occurred in the study [71]. The authors showed in a univariate analysis that anti-CTLA-4 therapy was associated with a higher risk of GI side effects compared to anti-PD-1/PD-L1 therapy (odds ratio 3.19; 95% CI 1.8–9.48; *p* = 0.037). In the multivariate analysis, the association could not be confirmed statistically; however, only 17% of the patients (*n* = 17) had received anti-CTLA-4 therapy at all [71]. Statistical significance was probably difficult to achieve due to the small number of patients. In fact, only 25% of the patients were able to undergo the complete treatment; 13% either died or were lost to follow-up, and 52% had progressive disease at the end of the ICI therapy [71].

In a recently published systematic review with meta-analysis including 193 patients from 12 studies, 39.8% patients (95% CI 26–55%) experienced relapse of their IBD when treated with an ICI [75]. In both the meta-analysis by Meserve et al. and the large retrospective study by Abu-Sbeih et al., approximately 75% of the IBD patients needed corticosteroids to treat the flare up [71,75]. The two studies differed in the administration of biologicals: these drugs were used in 28.6% of the patients with GI adverse events in the retrospective study [71]. In the meta-analysis (which included this retrospective study), they were necessary in 36.6% (95% CI 21.9–52.7%) and thus in approximately every second patient who was on corticosteroids due to the flare up [75]. However, the difference in percentages must be viewed with caution, as the other studies included in the meta-analysis evaluated far fewer patients than Abu-Sbeih et al.’s study, and therefore individual events in these smaller studies would have a greater impact on the perceived incidence [71,75]. In the meta-analysis, 35.4% of the patients (95% CI 16.8–56.7%) discontinued therapy with the ICI, but there was considerable heterogeneity between studies (I^2^ = 82%) [75].

According to the above and the previously described histopathological differences, IBD patients on ICI medication appear to be at risk for recurrence of colitis. However, the two forms of colitis in their acute form appear to be distinctly different to each other, both histologically and clinically, for example in terms of severity and potential for rapid progression of complications [33]. In addition, it is not yet clear whether chronic colitis on ICI therapy is similar to long-term IBD colitis [33].

### 4.4. ICI-Induced Colitis in the Elderly

Most prospective randomized trials did not perform subgroup analysis concerning differences between elderly and younger patients. The CheckMate-459 and KEYNOTE-240 trials showed equal efficacy of the study treatments in comparison to placebo in patients below 65 years of age and above 65 years for hepatocellular carcinoma (HCC) [7,76,77]. Comparable efficacy between younger and older patients was also seen in CTLA-4 therapy for melanoma [78,79,80], and with various ICI therapies in non-small cell lung cancer [81].

A recently published prospective observational study compared the two cohorts of under 70 years of age and over 70 years of age and found no differences in the rate of irAE development [82]. This is particularly interesting as the older cohort had significantly more comorbidities [82]. The rate of adverse events with ipilimumab in patients aged over 70 years was consistent with that observed elsewhere in studies with younger patients, with a similar incidence of grade 3–4 treatment-related adverse events [80].

However, another retrospective cohort study that examined the results in patients 75 years of age and over found that 23.7% of the elderly experienced grade 3 adverse events, and 24% had to stop treatment due to adverse events [83]. An exploratory analysis of phase 3 studies with a total of 1242 patients on nivolumab showed that the incidence of grade 1–2 adverse events did not differ between the cohort groups under 65 years (*n* = 616), over 65 years (*n* = 414), and over 70 years of age (*n* = 212) [84,85,86,87,88]. Of note, there were more serious adverse events in patients over 65 years and more serious toxicities (grade 3–5 adverse events) in patients over 70 years. In addition, it was evident that of the different irAEs, diarrhea and colitis in particular occurred more frequently in the older age groups (<65 years: 2.4%; >65 years: 4.1%; >70 years: 5.2%) [84]. Since elderly people are more susceptible to hypovolemia, the clinical consequences should not be underestimated.

## 5. Diagnosis

Patients on ICI therapy who develop diarrhea should undergo baseline investigations to exclude opportunistic infections (*Clostridium difficile* and other enteropathogens), which are the most important differential diagnosis, and measurement of serum inflammatory markers and electrolytes [89]. The most significant abnormalities are anemia, increased C-reactive protein, and low serum albumin levels [90]. A thyroid function test and cytomegalovirus PCR should also be performed [12]. In milder cases, rectoscopy might be indicated to confirm the diagnosis if symptoms do not improve or if the patient has already developed severe clinical symptoms, such as signs of peritonitis, at the initial presentation. This investigation should be done before starting systemic glucocorticoids [89]. In the vast majority of cases, the sigmoid colon and rectum are involved; flexible sigmoidoscopy is therefore generally sufficient to make the diagnosis [34,89,90]. Imaging should always be performed if serious complications are possible and need to be excluded. In general, as patients with colitis may deteriorate within days, the diagnosis should be made promptly and treatment initiated early [91].

## 6. Treatment

For treatment, guidelines from the European Society for Medical Oncology (ESMO) recommend a symptomatic approach in low-grade colitis with the substitution of oral fluids, a low-fiber diet, and loperamide [89]. At this stage, ICI therapy can be continued [89]. If the duration of symptoms is prolonged or if hematochezia develops, oral steroids should be given [12,89]. If symptoms worsen, ICI therapy should be discontinued, and systemic corticosteroids administered [89]. Patients who do not respond to steroids within a few days should receive infliximab [12,89]. While most patients recover with a single dose, some require a second dose of infliximab two weeks after the first administration [34,89,90,92]. Vedolizumab is also a reasonable option [91]. Although there are considerably less data, a systematic review showed equal efficacy of vedolizumab in comparison to infliximab [93]. Treatment with mycophenolate mofetil has also been described [94,95]. In most cases, these therapies can be discontinued within a reasonable period, but reports of treatment for an indefinite period due to persistent irAE have also been described [96].

### 6.1. Fecal Transplantation

The premise that stool transplantation can help ICI-induced colitis is that the composition of the microbiome has an impact on the occurrence and course of ICI-induced colitis. There are increasing data, primarily from animal studies, showing that the gut microbiome is involved in the development of ICI-induced colitis. In addition, it has been shown experimentally that the anti-tumor effect of ICIs can be reduced or enhanced by modulating the gut microbiome [12,31,97,98,99]. Furthermore, different intestinal microbiomes have been found to be associated with improvement or alternatively with the risk of developing ICI-induced colitis [29,100,101].

Knowledge of the therapeutic potential of fecal transplantation is therefore increasing, but the implementation into clinical use has only rarely been reported.

Wang et al. published the first two cases receiving fecal transplantation due to non-healing colitis as an irAE [99]. Both patients had complete recovery after treatment with the fecal transplant, although one of the patients needed two fecal transplants for complete resolution [99]. Fasanello et al. presented another case report in which fecal transplantation was successful in healing colitis [102].

### 6.2. Surgery

To date, surgery is a treatment of last resort for irAE colitis. An emergency colectomy is usually performed due to perforation or toxic megacolon [90]. A less aggressive therapeutic approach has occasionally been described and involves the formation of a defunctioning ileostomy without a large bowel resection in order to deviate the fecal stream instead of resecting the colon. In patients with severe acute IBD colitis, a colectomy is usually performed when conservative therapy fails. However, there may be room for construction of an ileostomy as an alternative in the emergency setting in selected cases in both CD and UC [103].

Sarnaik et al. reported on the creation of an ileostomy in a patient with therapy-refractory colitis due to ipilimumab therapy [104]. Our own group also showed resolution of a severe form of irAE colitis after construction of an ileostomy [105]. Therefore, in patients with severe colitis but without perforation, emergency formation of a defunctioning ileostomy may be an alternative to a colectomy. In such cases, ICI medication could be resumed much earlier than after colectomy.

### 6.3. Restarting Therapy

Although the efficacy of ICI therapy is now well established, there remains limited evidence on the safety profile of resuming ICI treatment in patients who have discontinued treatment due to development of irAE. In particular, there are still few data on how specific irAEs affect when ICI therapy can be restarted. A recent study used the World Health Organization’s VigiBase database to examine individual cases of reported irAEs and resumption of medication with the eventual outcomes [106]. Information on the restarting of medication and its consequences was available in 452 cases; 123 cases had colitis on initial ICI therapy [106]. A total of 37% (95% CI 29–45%) of these 123 patients developed colitis again after resumption of ICI therapy. Colitis as an adverse event showed a significantly higher risk of recurrence than other irAEs, such as adrenal diseases [106]. In the multivariate analysis, colitis and factors such as use of CTLA-4 medication, age, hepatitis, and pneumonitis were found to be significantly correlated with the risk of recurrence (OR 2.99; 95% CI 1.6–5.59; *p* < 0.001) [106].

A further study looked at cases where treatment with ICI was resumed after colitis had healed [107]. Initial ICI therapy was with a PD-1/PD-L1 inhibitor in just under half of patients (47%; *n* = 79), a CTLA-4 antagonist in 28% (*n* = 47), and both in a quarter of patients (*n* = 41). The vast majority (81%) of patients resumed ICI therapy with an anti-PD-1/PD-L1 agent, while 19% of patients resumed therapy with an anti-CTLA-4 agent [107]. Afterwards, diarrhea or colitis reoccurred in about one third of patients (*n* = 57); slightly more than half of these (54%; *n* = 30) developed grade 1 colitis [107]. In the case of recurrence of diarrhea or colitis, no difference in severity was observed between the two groups receiving anti-CTLA-4 or anti-PD-1/PD-L1 medication, but recurrence occurred significantly earlier after resumption of therapy with an anti-CTLA-4 drug. In addition, it was found that recurrent irAEs were more severe and required more intensive immunosuppressive therapy if immunosuppression had already been administered in the first episode [107]. Similarly, the patients who already received immunosuppressive therapy in the first episode had a higher risk of redeveloping diarrhea or colitis after resuming therapy [107]. Other studies have also described acceptable rates of toxicity after resuming ICI therapy. Therefore, it can be assumed that resuming treatment is acceptable, but close monitoring is required during this time [108,109].

## 7. Conclusions

Therapy with ICIs has fundamentally changed and improved oncological therapy, and thus the prognosis of countless patients. Since their mode of action is fundamentally different to classical chemotherapy, they are associated with a different spectrum of adverse events. Much of ICI-induced colitis is similar to IBD colitis and the drug therapy currently used is also equivalent. However, it is important to note that there may be room for new forms of therapy. Fecal transplantation and the creation of a defunctioning ileostomy should be considered earlier, as they are less aggressive than colectomy and ICI therapy can be restarted earlier. Since there are increasing data indicating a correlation between a favorable response and the development of colitis, it is especially important in these patients with colitis to continue ICI therapy for as long as possible.

## Figures and Tables

**Table 1:** Clinical characteristics of ICI-induced colitis. [14].

Colitis as Adverse Event					
	Grade 1	Grade 2	Grade 3	Grade 4	Grade 5
Clinical presentation	Asymptomatic (no intervention indicated)	Abdominal pain, mucus or blood in the stool	Severe abdominal pain, peritoneal signs	Life-threatening (perforation, ischemia, necrosis, bleeding, toxic megacolon); urgent intervention indicated	Death

**Table 2:** Immune checkpoint inhibitor therapy in patients with inflammatory bowel disease. CD: Crohn’s disease; CTLA-4: cytotoxic T-lymphocyte-associated antigen 4; IBD: inflammatory bowel disease; IC: indeterminate colitis; MD: microscopic colitis; PD-1: programmed cell death protein 1; PD-L1: programmed death ligand 1; n.r.: not reported; UC: ulcerative colitis.

Author	Year		Underlying Disease (Number of Patients)	Substance	Relapse IBD Colitis (Number of Patients)	Time to Flare-up	Grade 3 Colitis	Grade 4 Colitis	Permanent Discontinuation of ICI due to Immuno-Toxicity	Death
Braga Netoempty [72]	2020	Case series	IBD (13) CD (5) CU (8)	Pembrolizumab Nivolumab Ipilimumab (Percentages n.r.)	CD 20% (1) UC 37.5% (3) With ipilimumab: 9.1% With PD-1: 1.6%	Median 5 months (range n.r.)	n.r.	n.r.	0	n.r.
Grover [73]	2020	Cohort	IBD (21) MC (4) CD (10) UC (9) IC (1)	Monotherapy: PD-1/PD-L1 (88%) Ipilimumab (4%) Combination of Ipilimumab & Nivolumab (8%)	IBD 19% (4) (UC 3, IC 1) MC 75% (3)	Median 7 weeks (range 4–40 weeks)	*n* = 8		*n* = 6	n.r.
Abu-Sbeih [71]	2020	Cohort	IBD (102) CD (49) UC (49) Unclassified IBD (4)	PD-1/PD-L1 (83%) CTLA-4 (7%) Combination (10%)	36%	Mean 62 days (IQR 33–123 days)	14%	3%	n.r.	0
Iwamoto [74]	2020	Case report	UC (1)	Nivolumab	Yes	5 months	-	-	1	n.r
Tison [70]	2019	Cohort Studies	Total *n* = 112 Most frequent: Psoriasis (31) Rheumatoid arthritis (20) IBD (14) Lupus (7)	Monotherapy: Nivolumab Pembrolizumab Ipilimumab Atezolizumab Avelumab Combination of ipilimumab & nivolumab	50% of IBD patients	n.r.	86% of IBD patients with flare-up		36% of IBD patients with flare-up	1
Meserve [75]	2020	Systematic review and meta-analysis	IBD (193)	PD-1/PD-L1 (*n* = 149) CTLA-4 (*n* = 22) Combination (*n* = 22)	40% patients (95% CI 26–55%)	Could not be calculated	-	-	35.4% patients (95% CI 16.8–56.7%)	0

## Data Availability

Not applicable.

## Supplementary Materials

The following are available online at https://www.mdpi.com/article/10.3390/ph14040367/s1, Table S1: Colitis under therapy with nivolumab, Table S2: Colitis under therapy with ipilimumab, Table S3: Colitis under therapy with nivolumab in combination with ipilimumab, Table S4: Colitis under therapy with durvalumab alone or in combination with tremelimumab, Table S5: Colitis under therapy with azetolizumab alone or in different combinations, Table S6: Colitis under therapy with different combinations of ICI agents.
