# Supplementary material for: Checkpoint Inhibitor-Induced Colitis—A Clinical Overview of Incidence, Prognostic Implications and Extension of Current Treatment Options
Source: Pharmaceuticals (Basel). 2021 Apr 16;14(4):367. doi: 10.3390/ph14040367 (PMC8074139; doi:10.3390/ph14040367)
Supplement: Supplementary file 1 [file pharmaceuticals-14-00367-s001.zip › pharmaceuticals-1163593-SI.pdf]

Supplementary Tables:
Colitis rates in prospective studies with different ICI agents

Author	Year	n	Underlying disease	Substance	all over rate colitis	Grade 1	Grade 2	Grade 3	Grade 4
**Fukuoka [1]**	2020	50	gastric and colorectal cancer	CTX previously Nivolumab + Regorafenib (**A** 80mg; **B** 120mg, **C** 160 mg)	n.r.<br>Diarrhoea 22%			Diarrhoea<br>**A** 0% vs. **B** 4% vs. **C** 0%	
**Feng [2]**	2020	32	met. biliary tract cancer	Nivolumab + Gemcitabine + cisplatin	n.r.				
**Kato [3]**	2020	209	oesophageal cancer	**A** Nivolumab vs.<br>**B** Chemotherapy	n.r.	Diarrhoea<br>**A** 10% (n=20)<br>**B** 9% (n=18)		Diarrhoea<br>**A** 1% (n=2)<br>**B** 1% (n=2)	
**Mc Farlane [4]**	2020	97	clear cell renal carcinoma	Nivolumab	n.r.				
**Reardon [5]**	2020	369	glioblastoma	**A** Nivolumab vs.<br>**B** Bevacizumab	n.r.<br>Diarrhoea<br>**A** 5.5%<br>**B** 1.2%				
**Peters [6]**	2019	82	non small cell lung cancer	Nivolumab + CTX (platin based) + radiotherapy	1.3% (n=1)				**Grade 5**<br>1.3% (n=1)
**Ueno [7]**	2019	60	biliary tract cancer	**A** Nivolumab vs.<br>**B** Nivolumab + GemCis	n.r.	Diarrhoea<br>**A** 7%<br>**B** 7%			
**Overman [8]**	2017	74	MSI CRC	Nivolumab	1% (n= 1)			1% (n= 1)	
**Larkin [9]**	2015	945	melanoma	**A** Nivolumab vs.<br>**B** Nivolumab + Ipilimumab vs.<br>**C** Ipilimumab	**A** 1.3%<br>**B** 11.8%<br>**C** 11.6%			**A** 0.6%<br>**B** 7.7%<br>**C** 8.7%	

Supplementary Table 1a: Colitis under therapy with nivolumab. In case colitis was not reported but diarrhoea, we report in all tables the percentage of diarrhoea; n.r.: not reported; MSI: microsatellite-instable; CRC: colorectal cancer

Supplementary Tables:
Colitis rates in prospective studies with different ICI agents

Author	Year	n	Underlying disease	Substance	all over rate colitis	Grade 1	Grade 2	Grade 3	Grade 4
**Ascierto [10]**	2017	727	melanoma	**A** Ipilimumab 3mg/kg KG		3% (n=10)		2% (n=9)	0%
				**B** Ipilimumab 10mg/kg KG		4% (n=16)		5% (n=18)	1% (n=1)
**Eggermont [11]**	2016	951	melanoma	Ipilimumab 10mg/kg KG	15.5 % (n=73)			6.8% (n=32)	0.8% (n=4)<br>**Grade 5**<br>0.6% (n=3)
**Yamazaki [12]**	2015	15	melanoma	Ipilimumab + Dacarbazin	6.7% (n=1)	0	6.7% (n=1)	0	0
**Luke [13]**	2015	32	melanoma	Ipilimumab 3mg/kg KG + GM-CSF	7.4% n=2			7.4% n=2	
**Hodi [14]**	2014	A 118<br><br>B 120	Met. melanoma	**A** Ipilimumab 10mg/kg KG + GM-CSF<br>**B** Ipilimumab 10mg/kg KG				**Grade 3-5**<br>A 5.9% n=7<br><br>B 8.3% n=10	
**Margolin [15]**	2012	72	Brain metastasis of melanoma	Ipilimumab	n.r.	n.r.	n.r.	n.r.	Grade 5 n=1

Supplementary Table 1b: Colitis under therapy with ipilimumab; GM-CSF: granulocyte-macrophage colony-stimulating factor

Supplementary Tables:
Colitis rates in prospective studies with different ICI agents

Author	Year	n	Underlying disease	Substance	all over rate colitis	Grade 1	Grade 2	Grade 3	Grade 4
**Montfort [16]**	2021	14	melanoma	Nivolumab + Ipilimumab<br>**A** +/- Certoluzimab<br>**B** +/- Infliximab		0	0 vs. 1	0 vs. 1	0
**Kleef [17]**	2020	131	advanced disease	low-dose Ipilimumab + Nivolumab in combination with IL-2 + hyperthermia				1.5% (n=2)	
**Sharma [18]**	2020	90	met. Castration-resistant prostate cancer	**A** Ipilimumab + Nivolumab pre- Chemotherapy	8.9% (n=4)			4.4% (n=2)	
				**B** Ipilimumab + Nivolumab post Chemotherapy	17.8% (n=8)			11.1% (n=5)	
**Gao [19]**	2020	28	urothelial carcinoma	Durvalumab + Tremelimumab	11% (n=3)			4% (n=1)	
**Hodi [20]**	2018	A 313<br><br>B 313<br>C 311	melanoma	**A** Nivolumab + Ipilimumab<br>**B** Nivolumab alone<br>**C** Ipilimumab alone		A 5% n=14<br><br>B 2% n=5<br>C 4% n=11		A 8% (n=25)<br><br>B 1% (n=3)<br>C 7% (n=23)	A <1% (n=1)<br>B 0<br>C <1% (n=1)
**Weber [21]**	2016	140	melanoma	**A** 1st Nivolumab<br>2nd Ipilimumab		3% (n=1)			
				**B** 1st Ipilimumab<br>2nd Nivolumab				3% (n=1)	
**Hodi [22]**	2016	A 94<br><br>B 47	advanced melanoma	**A** Nivolumab + Ipilimumab<br>**B** Ipilimumab alone	11% (n=10)<br><br>B 2% (n=1)	A 5% (n=5)<br><br>B 4% (n=2)		A 12% (n=11)<br><br>B 2% (n=1)	A 0<br><br>B 0

Supplementary Table 1c: Colitis under therapy with nivolumab in combination with ipilimumab

Supplementary Tables:
Colitis rates in prospective studies with different ICI agents

Author	Year	n	Underlying disease	Substance	all over rate colitis	Grade 1 and Grade 2	Grade 3	Grade 4
**Boilêve [23]**	2021	20	biliary tract carcinoma	**A** Durvalumab + Tremelimumab<br>**B** Durvalumab + Tremelimumab + Paclitaxel		**A** 10% (n=1)<br>**B** 10% (n=1)	**B** 20% (n=2)	
**Nowak [24]**	2020	54	pleural mesothelioma	Durvalumab + CTX (Cisplatin+ Pemetrexed)	n.r.	Diarrhoea 24%	Diarrhoea 2%	
**Powles [25]**	2020	1032	urothelial carcinoma	**A** Durvalumab vs.<br>**B** Durvalumab +Tremelimumab		**A** 1 % (n=3)<br>**B** 2 % (n=8)	**B** 2% (n=7)	
**Garassino [26]**	2018	444	non-small cell lung cancer	Durvalumab		1% (n=2)		
**Sarfaty [27]**	2020	13	met. non-urothelial urinary tract carcinoma	Durvalumab + Tremelimumab	n.r.	Diarrhoea 7.6%	Diarrhoea 15.3%	
**Chen [28]**	2020	180	advanced CRC	**A** Durvalumab + Tremelimumab vs<br>**B** best supportive care	n.r. Diarrhoea<br>**A** 31% (n=37)<br>**B** 10% (n=6)		Diarrhoea<br>**A** 4% (n=5)	

Supplementary Table 1d: Colitis under therapy with durvalumab alone or in combination with tremelimumab

Supplementary Tables:
Colitis rates in prospective studies with different ICI agents

Author	Year	n	Underlying disease	Substance	all over rate colitis	Grade 1 and Grade 2	Grade 3	Grade 4
**Gogas [29]**	2020	446	melanoma	**A** Azetolizumab + cobimetinib vs. **B** pembrolizumab	Diarrhoea **A** 54.1% **B** 16.2%		Diarrhoea **A** 7.7% ; **B** 1.9%	
**Rosenberg [30]**	2016	310	urothelial carcinoma	Azetolizumab	1% (n=3)		1% (n=2)	
**Gutzmer [31]**	2020	511	melanoma BRAF mutation positive	**A** Azetolizumab + cobimetinib + Vemurafenib vs. **B** Placebo + Vemurafenib + cobimetinib	n.r. Diarrhoea **A** 42% (n=97) **B** 47% (n=131)		Diarrhoea **A** 2% (n=4) **B** 3% (n=9)	
**Shu [32]**	2020	30	non-small cell lung cancer	Atezolizumab + CTX in a neoadjuvant setting	n.r. Diarrhoea 30% (n=8)		Diarrhoea 3% (n=1)	

Supplementary Table 1e: Colitis under therapy with Azetolizumab alone or in different combinations

Supplementary Tables:
Colitis rates in prospective studies with different ICI agents

Author	Year	n	Underlying disease	Substance	all over rate colitis	Grade 1	Grade 2	Grade 3	Grade 4
**Friedlander [33]**	2019	49	solid tumours	Pamiparib + Tislelizumab (5 dose escalation cohorts)	n.r.	Diarrhoea 35% (n=17)		Diarrhoea 4% (n=2)	
**Barlesi [34]**	2018	792	non-small cell lung cancer	**A** Avelumab vs.<br>**B** Docetaxel	n.r.	**A** Diarrhoea 6% (n=24)<br>**B** Diarrhoea 14% (n=51)		Diarrhoea **B** 1% (n=4)	Diarrhoea <1% (n=1)
**Schmid [35]**	2020	60	triple-negative breast cancer	Pembrolizumab + chemotherapy	5% (n=3)		1.7% (n=1)	3% (n=2)	
**Taylor [36]**	2020	137	solid tumours	Pembrolizumab + chemotherapy + Lenvantinib	4% (n=5)			2% (n=2)	
**Tolaney [37]**	2020	88	breast cancer	A Pembrolizumab + Eribulin vs.<br>B Eribulin	5% (n=2)				grade 5 lethal: 5% (n=2)

Supplementary Table 1f: Colitis under therapy with different combinations of ICI agents

Supplementary Tables:
Colitis rates in prospective studies with different ICI agents

References

1. Fukuoka, S.; Hara, H.; Takahashi, N.; Kojima, T.; Kawazoe, A.; Asayama, M.; Yoshii, T.; Kotani, D.; Tamura, H.; Mikamoto, Y., et al. Regorafenib Plus Nivolumab in Patients With Advanced Gastric or Colorectal Cancer: An Open-Label, Dose-Escalation, and Dose-Expansion Phase Ib Trial (REGONIVO, EPOC1603). *J Clin Oncol* **2020**, *38*, 2053-2061, doi:10.1200/JCO.19.03296.
2. Feng, K.; Liu, Y.; Zhao, Y.; Yang, Q.; Dong, L.; Liu, J.; Li, X.; Zhao, Z.; Mei, Q.; Han, W. Efficacy and biomarker analysis of nivolumab plus gemcitabine and cisplatin in patients with unresectable or metastatic biliary tract cancers: results from a phase II study. *J Immunother Cancer* **2020**, *8*, doi:10.1136/jitc-2019-000367.
3. Kato, K.; Cho, B.C.; Takahashi, M.; Okada, M.; Lin, C.Y.; Chin, K.; Kadowaki, S.; Ahn, M.J.; Hamamoto, Y.; Doki, Y., et al. Nivolumab versus chemotherapy in patients with advanced oesophageal squamous cell carcinoma refractory or intolerant to previous chemotherapy (ATTRACTION-3): a multicentre, randomised, open-label, phase 3 trial. *Lancet Oncol* **2019**, *20*, 1506-1517, doi:10.1016/S1470-2045(19)30626-6.
4. McFarlane, J.J.; Kochenderfer, M.D.; Olsen, M.R.; Bauer, T.M.; Molina, A.; Hauke, R.J.; Reeves, J.A.; Babu, S.; Van Veldhuizen, P.; Somer, B., et al. Safety and Efficacy of Nivolumab in Patients With Advanced Clear Cell Renal Cell Carcinoma: Results From the Phase IIIb/IV CheckMate 374 Study. *Clin Genitourin Cancer* **2020**, *18*, 469-476 e464, doi:10.1016/j.clgc.2020.06.002.
5. Reardon, D.A.; Brandes, A.A.; Omuro, A.; Mulholland, P.; Lim, M.; Wick, A.; Baehring, J.; Ahluwalia, M.S.; Roth, P.; Bahr, O., et al. Effect of Nivolumab vs Bevacizumab in Patients With Recurrent Glioblastoma: The CheckMate 143 Phase 3 Randomized Clinical Trial. *JAMA Oncol* **2020**, *6*, 1003-1010, doi:10.1001/jamaoncol.2020.1024.
6. Peters, S.; Felip, E.; Dafni, U.; Belka, C.; Guckenberger, M.; Irigoyen, A.; Nadal, E.; Becker, A.; Vees, H.; Pless, M., et al. Safety evaluation of nivolumab added concurrently to radiotherapy in a standard first line chemo-radiotherapy regimen in stage III non-small cell lung cancer-The ETOP NICOLAS trial. *Lung Cancer* **2019**, *133*, 83-87, doi:10.1016/j.lungcan.2019.05.001.
7. Ueno, M.; Ikeda, M.; Morizane, C.; Kobayashi, S.; Ohno, I.; Kondo, S.; Okano, N.; Kimura, K.; Asada, S.; Namba, Y., et al. Nivolumab alone or in combination with cisplatin plus gemcitabine in Japanese patients with unresectable or recurrent biliary tract cancer: a non-randomised, multicentre, open-label, phase 1 study. *Lancet Gastroenterol Hepatol* **2019**, *4*, 611-621, doi:10.1016/S2468-1253(19)30086-X.
8. Overman, M.J.; McDermott, R.; Leach, J.L.; Lonardi, S.; Lenz, H.J.; Morse, M.A.; Desai, J.; Hill, A.; Axelson, M.; Moss, R.A., et al. Nivolumab in patients with metastatic DNA mismatch repair-deficient or microsatellite instability-high colorectal cancer (CheckMate 142): an open-label, multicentre, phase 2 study. *Lancet Oncol* **2017**, *18*, 1182-1191, doi:10.1016/S1470-2045(17)30422-9.
9. Larkin, J.; Chiarion-Sileni, V.; Gonzalez, R.; Grob, J.J.; Cowey, C.L.; Lao, C.D.; Schadendorf, D.; Dummer, R.; Smylie, M.; Rutkowski, P., et al. Combined Nivolumab and Ipilimumab or Monotherapy in Untreated Melanoma. *N Engl J Med* **2015**, *373*, 23-34, doi:10.1056/NEJMoa1504030.
10. Ascierto, P.A.; Del Vecchio, M.; Robert, C.; Mackiewicz, A.; Chiarion-Sileni, V.; Arance, A.; Lebbe, C.; Bastholt, L.; Hamid, O.; Rutkowski, P., et al. Ipilimumab 10 mg/kg versus ipilimumab 3 mg/kg in patients with unresectable or metastatic melanoma: a randomised, double-blind, multicentre, phase 3 trial. *Lancet Oncol* **2017**, *18*, 611-622, doi:10.1016/S1470-2045(17)30231-0.

11. Eggermont, A.M.; Chiarion-Sileni, V.; Grob, J.J.; Dummer, R.; Wolchok, J.D.; Schmidt, H.; Hamid, O.; Robert, C.; Ascierto, P.A.; Richards, J.M., et al. Prolonged Survival in Stage III Melanoma with Ipilimumab Adjuvant Therapy. *N Engl J Med* **2016**, *375*, 1845-1855, doi:10.1056/NEJMoa1611299.
12. Yamazaki, N.; Uhara, H.; Fukushima, S.; Uchi, H.; Shibagaki, N.; Kiyohara, Y.; Tsutsumida, A.; Namikawa, K.; Okuyama, R.; Otsuka, Y., et al. Phase II study of the immune-checkpoint inhibitor ipilimumab plus dacarbazine in Japanese patients with previously untreated, unresectable or metastatic melanoma. *Cancer chemotherapy and pharmacology* **2015**, *76*, 969-975, doi:10.1007/s00280-015-2870-0.
13. Luke, J.J.; Donahue, H.; Nishino, M.; Giobbie-Hurder, A.; Davis, M.; Bailey, N.; Ott, P.A.; Hodi, F.S. Single Institution Experience of Ipilimumab 3 mg/kg with Sargramostim (GM-CSF) in Metastatic Melanoma. *Cancer Immunol Res* **2015**, *3*, 986-991, doi:10.1158/2326-6066.CIR-15-0066.
14. Hodi, F.S.; Lee, S.; McDermott, D.F.; Rao, U.N.; Butterfield, L.H.; Tarhini, A.A.; Leming, P.; Puzanov, I.; Shin, D.; Kirkwood, J.M. Ipilimumab plus sargramostim vs ipilimumab alone for treatment of metastatic melanoma: a randomized clinical trial. *JAMA* **2014**, *312*, 1744-1753, doi:10.1001/jama.2014.13943.
15. Margolin, K.; Ernstoff, M.S.; Hamid, O.; Lawrence, D.; McDermott, D.; Puzanov, I.; Wolchok, J.D.; Clark, J.I.; Sznol, M.; Logan, T.F., et al. Ipilimumab in patients with melanoma and brain metastases: an open-label, phase 2 trial. *Lancet Oncol* **2012**, *13*, 459-465, doi:10.1016/S1470-2045(12)70090-6.
16. Montfort, A.; Filleron, T.; Virazels, M.; Dufau, C.; Milhes, J.; Pages, C.; Olivier, P.; Ayyoub, M.; Mounier, M.; Lusque, A., et al. Combining Nivolumab and Ipilimumab with Infliximab or Certolizumab in Patients with Advanced Melanoma: First Results of a Phase Ib Clinical Trial. *Clin Cancer Res* **2021**, *27*, 1037-1047, doi:10.1158/1078-0432.CCR-20-3449.
17. Kleef, R.; Nagy, R.; Baierl, A.; Bacher, V.; Bojar, H.; McKee, D.L.; Moss, R.; Thoennissen, N.H.; Szasz, M.; Bakacs, T. Low-dose ipilimumab plus nivolumab combined with IL-2 and hyperthermia in cancer patients with advanced disease: exploratory findings of a case series of 131 stage IV cancers - a retrospective study of a single institution. *Cancer Immunol Immunother* **2020**, 10.1007/s00262-020-02751-0, doi:10.1007/s00262-020-02751-0.
18. Sharma, P.; Pachynski, R.K.; Narayan, V.; Flechon, A.; Gravis, G.; Galsky, M.D.; Mahammedi, H.; Patnaik, A.; Subudhi, S.K.; Ciprotti, M., et al. Nivolumab Plus Ipilimumab for Metastatic Castration-Resistant Prostate Cancer: Preliminary Analysis of Patients in the CheckMate 650 Trial. *Cancer Cell* **2020**, *38*, 489-499 e483, doi:10.1016/j.ccell.2020.08.007.
19. Gao, J.; Navai, N.; Alhalabi, O.; Siefker-Radtke, A.; Campbell, M.T.; Tidwell, R.S.; Guo, C.C.; Kamat, A.M.; Matin, S.F.; Araujo, J.C., et al. Neoadjuvant PD-L1 plus CTLA-4 blockade in patients with cisplatin-ineligible operable high-risk urothelial carcinoma. *Nat Med* **2020**, *26*, 1845-1851, doi:10.1038/s41591-020-1086-y.
20. Hodi, F.S.; Chiarion-Sileni, V.; Gonzalez, R.; Grob, J.J.; Rutkowski, P.; Cowey, C.L.; Lao, C.D.; Schadendorf, D.; Wagstaff, J.; Dummer, R., et al. Nivolumab plus ipilimumab or nivolumab alone versus ipilimumab alone in advanced melanoma (CheckMate 067): 4-year outcomes of a multicentre, randomised, phase 3 trial. *Lancet Oncol* **2018**, *19*, 1480-1492, doi:10.1016/S1470-2045(18)30700-9.
21. Weber, J.S.; Gibney, G.; Sullivan, R.J.; Sosman, J.A.; Slingluff, C.L., Jr.; Lawrence, D.P.; Logan, T.F.; Schuchter, L.M.; Nair, S.; Fecher, L., et al. Sequential administration of nivolumab and ipilimumab with a planned switch in patients with advanced melanoma (CheckMate 064): an open-label, randomised, phase 2 trial. *Lancet Oncol* **2016**, *17*, 943-955, doi:10.1016/S1470-2045(16)30126-7.
22. Hodi, F.S.; Chesney, J.; Pavlick, A.C.; Robert, C.; Grossmann, K.F.; McDermott, D.F.; Linette, G.P.; Meyer, N.; Giguere, J.K.; Agarwala, S.S., et al. Combined nivolumab and ipilimumab

versus ipilimumab alone in patients with advanced melanoma: 2-year overall survival outcomes in a multicentre, randomised, controlled, phase 2 trial. *Lancet Oncol* **2016**, *17*, 1558-1568, doi:10.1016/S1470-2045(16)30366-7.
23. Boileve, A.; Hilmi, M.; Gougis, P.; Cohen, R.; Rousseau, B.; Blanc, J.F.; Ben Abdelghani, M.; Castanie, H.; Dahan, L.; Tougeron, D., et al. Triplet combination of durvalumab, tremelimumab, and paclitaxel in biliary tract carcinomas: Safety run-in results of the randomized IMMUNOBIL PRODIGE 57 phase II trial. *Eur J Cancer* **2021**, *143*, 55-63, doi:10.1016/j.ejca.2020.10.027.
24. Nowak, A.K.; Lesterhuis, W.J.; Kok, P.S.; Brown, C.; Hughes, B.G.; Karikios, D.J.; John, T.; Kao, S.C.; Leslie, C.; Cook, A.M., et al. Durvalumab with first-line chemotherapy in previously untreated malignant pleural mesothelioma (DREAM): a multicentre, single-arm, phase 2 trial with a safety run-in. *Lancet Oncol* **2020**, *21*, 1213-1223, doi:10.1016/S1470-2045(20)30462-9.
25. Powles, T.; van der Heijden, M.S.; Castellano, D.; Galsky, M.D.; Loriot, Y.; Petrylak, D.P.; Ogawa, O.; Park, S.H.; Lee, J.L.; De Giorgi, U., et al. Durvalumab alone and durvalumab plus tremelimumab versus chemotherapy in previously untreated patients with unresectable, locally advanced or metastatic urothelial carcinoma (DANUBE): a randomised, open-label, multicentre, phase 3 trial. *Lancet Oncol* **2020**, *21*, 1574-1588, doi:10.1016/S1470-2045(20)30541-6.
26. Garassino, M.C.; Cho, B.C.; Kim, J.H.; Mazieres, J.; Vansteenkiste, J.; Lena, H.; Corral Jaime, J.; Gray, J.E.; Powderly, J.; Chouaid, C., et al. Durvalumab as third-line or later treatment for advanced non-small-cell lung cancer (ATLANTIC): an open-label, single-arm, phase 2 study. *Lancet Oncol* **2018**, *19*, 521-536, doi:10.1016/S1470-2045(18)30144-X.
27. Sarfaty, M.; Whiting, K.; Teo, M.Y.; Lee, C.H.; Peters, V.; Durocher, J.; Regazzi, A.M.; McCoy, A.S.; Hettich, G.; Jungbluth, A.A., et al. A phase II trial of durvalumab and tremelimumab in metastatic, non-urothelial carcinoma of the urinary tract. *Cancer Med* **2020**, 10.1002/cam4.3699, doi:10.1002/cam4.3699.
28. Chen, E.X.; Jonker, D.J.; Loree, J.M.; Kennecke, H.F.; Berry, S.R.; Couture, F.; Ahmad, C.E.; Goffin, J.R.; Kavan, P.; Harb, M., et al. Effect of Combined Immune Checkpoint Inhibition vs Best Supportive Care Alone in Patients With Advanced Colorectal Cancer: The Canadian Cancer Trials Group CO.26 Study. *JAMA Oncol* **2020**, *6*, 831-838, doi:10.1001/jamaoncol.2020.0910.
29. Gogas, H.; Dreno, B.; Larkin, J.; Demidov, L.; Stroyakovskiy, D.; Eroglu, Z.; Francesco Ferrucci, P.; Pigozzo, J.; Rutkowski, P.; Mackiewicz, J., et al. Cobimetinib plus atezolizumab in BRAF(V600) wild-type melanoma: primary results from the randomized phase III IMspire170 study. *Ann Oncol* **2020**, 10.1016/j.annonc.2020.12.004, doi:10.1016/j.annonc.2020.12.004.
30. Rosenberg, J.E.; Hoffman-Censits, J.; Powles, T.; van der Heijden, M.S.; Balar, A.V.; Necchi, A.; Dawson, N.; O'Donnell, P.H.; Balmanoukian, A.; Loriot, Y., et al. Atezolizumab in patients with locally advanced and metastatic urothelial carcinoma who have progressed following treatment with platinum-based chemotherapy: a single-arm, multicentre, phase 2 trial. *Lancet* **2016**, *387*, 1909-1920, doi:10.1016/S0140-6736(16)00561-4.
31. Gutzmer, R.; Stroyakovskiy, D.; Gogas, H.; Robert, C.; Lewis, K.; Protsenko, S.; Pereira, R.P.; Eigentler, T.; Rutkowski, P.; Demidov, L., et al. Atezolizumab, vemurafenib, and cobimetinib as first-line treatment for unresectable advanced BRAF(V600) mutation-positive melanoma (IMspire150): primary analysis of the randomised, double-blind, placebo-controlled, phase 3 trial. *Lancet* **2020**, *395*, 1835-1844, doi:10.1016/S0140-6736(20)30934-X.
32. Shu, C.A.; Gainor, J.F.; Awad, M.M.; Chiuzan, C.; Grigg, C.M.; Pabani, A.; Garofano, R.F.; Stoopler, M.B.; Cheng, S.K.; White, A., et al. Neoadjuvant atezolizumab and chemotherapy in patients with resectable non-small-cell lung cancer: an open-label, multicentre, single-arm, phase 2 trial. *Lancet Oncol* **2020**, *21*, 786-795, doi:10.1016/S1470-2045(20)30140-6.

33. Friedlander, M.; Meniawy, T.; Markman, B.; Mileshkin, L.; Harnett, P.; Millward, M.; Lundy, J.; Freimund, A.; Norris, C.; Mu, S., et al. Pamiparib in combination with tislelizumab in patients with advanced solid tumours: results from the dose-escalation stage of a multicentre, open-label, phase 1a/b trial. *Lancet Oncol* **2019**, *20*, 1306-1315, doi:10.1016/S1470-2045(19)30396-1.
34. Barlesi, F.; Vansteenkiste, J.; Spigel, D.; Ishii, H.; Garassino, M.; de Marinis, F.; Ozguroglu, M.; Szczesna, A.; Polychronis, A.; Uslu, R., et al. Avelumab versus docetaxel in patients with platinum-treated advanced non-small-cell lung cancer (JAVELIN Lung 200): an open-label, randomised, phase 3 study. *Lancet Oncol* **2018**, *19*, 1468-1479, doi:10.1016/S1470-2045(18)30673-9.
35. Schmid, P.; Salgado, R.; Park, Y.H.; Munoz-Couselo, E.; Kim, S.B.; Sohn, J.; Im, S.A.; Foukakis, T.; Kuemmel, S.; Dent, R., et al. Pembrolizumab plus chemotherapy as neoadjuvant treatment of high-risk, early-stage triple-negative breast cancer: results from the phase 1b open-label, multicohort KEYNOTE-173 study. *Ann Oncol* **2020**, *31*, 569-581, doi:10.1016/j.annonc.2020.01.072.
36. Taylor, M.H.; Lee, C.H.; Makker, V.; Rasco, D.; Dutcus, C.E.; Wu, J.; Stepan, D.E.; Shumaker, R.C.; Motzer, R.J. Phase IB/II Trial of Lenvatinib Plus Pembrolizumab in Patients With Advanced Renal Cell Carcinoma, Endometrial Cancer, and Other Selected Advanced Solid Tumors. *J Clin Oncol* **2020**, *38*, 1154-1163, doi:10.1200/JCO.19.01598.
37. Tolaney, S.M.; Barroso-Sousa, R.; Keenan, T.; Li, T.; Trippa, L.; Vaz-Luis, I.; Wulf, G.; Spring, L.; Sinclair, N.F.; Andrews, C., et al. Effect of Eribulin With or Without Pembrolizumab on Progression-Free Survival for Patients With Hormone Receptor-Positive, ERBB2-Negative Metastatic Breast Cancer: A Randomized Clinical Trial. *JAMA Oncol* **2020**, *6*, 1598-1605, doi:10.1001/jamaoncol.2020.3524.
